# Novel COVID-19 Intersections with Dentistry: Approaches to protection

**DOI:** 10.4317/jced.57307

**Published:** 2021-04-01

**Authors:** Silvia Lourenço, João-Vitor Lopes, Gustavo-Henrique Boog, Lucas Chinelatto, Flavio Hojaij

**Affiliations:** 1 DDS, PHD. Associate Professor – Pathology Department - Dental School – University of São Paulo, Brazil; 2 Student – Faculty of Medicine – University of São Paulo, Brazil; 3 MD, PHD. Associate Professor – Surgery Department – Faculty of Medicine of the University of São Paulo, Brazil

## Abstract

**Background:**

COVID-19 outbreak brought many challenges to the society and to health care systems. Health care professionals in dental practices are a high-risk population, due to close contact to saliva and aerosols. Specific guidance for those professionals is essential to control disease transmission and guarantee dentist’s health.

**Material and Methods:**

We performed a literature review based on online search in Pubmed, Scientific Electronic Library, Literatura Latino-Americana e do Caribe em Ciências da Saúde and Scopus databases. Included articles were fully read and analysed regarding infection control measurements, use of personal protective equipment (PPE), conditions for procedures and use of mouthrinse.

**Results:**

Nineteen articles were added to this review. The majority of them recommended maintaining only urgency treatments, with use of rubber dam and mouthrinse before procedures.

**Conclusions:**

The data collected suggests dental care should be performed under strict protection measures such as a pre-check questionnaire prior to any dental procedure; postponing elective dental treatments and following infection control security procedures may be strategic at this point. The use of adequate PPE for procedures is recommended. Supplementary measures should rise from further understanding of the pandemics.

** Key words:**COVID-19, dentistry, practice.

## Introduction

The pandemic of the novel coronavirus-19 originated from an outbreak in Wuhan, China, has spread to all continents, and became an urgent public health challenge worldwide. Countries around the world have taken several measures to constrain the infection rates, and the recommendation of social distancing by the WHO has been followed as a public policy by most governments. Impacts on the many facets of society are still to be evaluated; activities in close inter-person contact are the most affected (1,2). In Brazil the contamination is still rising at this point and thriving concerns on procedures to protect dental practitioners and to avoid cross contamination are being discussed.

COVID-19 has been reputed as highly contagious and its transmittable forms are not fully understood. Mainly, it spreads from person to person, primarily by respiratory and saliva droplets (perdigottes), as well as through infected blood and body fluids, infected surfaces, amongst others. In this scenario, healthcare personnel at dental practices are at major risk; additionally, the risk of cross infection due to other causes is well known in the practice of dentistry. The teaching and practice of dentistry has been impacted by many manners, as Dental schools worldwide have to re approach their methods of training students and well-established professionals struggle with the many new guidelines to dental practice in private offices (1-4).

Dental Councils as well as sanitary authorities in countries around the 5 Continents have been discussing on the best forms to provide dental care with minimal impact to patients’ health and the dental team. Some believe that the dental professionals are used to deal with a contaminated environment and with cross infection topic, therefore, they could also deal with COVID-19 infection issues. Others believe in reducing the routine oral and dental care to avoid contamination as a moral duty, including Brazilian health authorities, who recommended dental care limited to urgency cases (3-6).

Therefore, information, guidelines and determination of strict protocols to control infections are important tools to aid dental practitioners, dental nurses, tutors and students to avoid hesitant measures and vulnerabilities for both professionals and patients (1-3).

We herein revise the main publications on COVID-19 to understand the boundaries between COVID-19 and the practice of dentistry.

## Material and Methods

Literature review was based on online search in PubMed from National Center for Biotechnology (NCBI), Scientific Electronic Library Online (Scielo), Literatura Latino-Americana e do Caribe em Ciências da Saúde (LILACS) and Scopus databases. The following terms were used in the search engine for any match in articles: ((COVID) OR (CORONAVIRUS) OR (SARS-COV-2)) AND ((DENTISTRY) OR (DENTAL) OR (PERIODONTOLOGY) OR (TONGUE) OR (GINGIVA)).

Articles included in the search comprised research, letters, reviews, editorials and internet databases if their publication was in the year of 2020 and their title was relevant to this article objectives. For the present review a reference was considered relevant if: (a) it was correlated with the COVID-19 pandemic; (b) it had an approach that directly impacted in dental practice dynamics; (c) it suggested any kind of procedures for professional to avoid SARS-CoV-2 infection (d); it suggested any way to evaluate patients admission or to protect patients in dentistry offices; (e) it had any kind of recommendation about elective procedures; (f) it made any consideration for dental practices reorganization regarding the pandemic context.

Duplicates were manually deleted. After that, all selected references were read in full extent, and added or not to the review after author consensus on its relevance. Articles that were not in English were translated using validated websites.

The references were analysed regarding the following topics: reference kind, infection control measures in the dental office, personal protective equipment (PPE) for dentists, patient evaluation methods, conditions for procedures and use of mouthrinse.

Data analysis was performed using the Google Sheets application (GOOGLE INC.).

## Results

**Figure 1:**
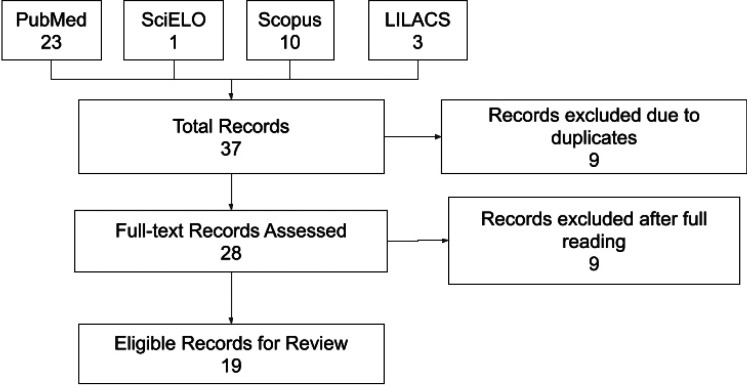
Inclusion Diagram. Scielo=Scientific Electronic Library Online LILACS= Literatura Latino-Americana e do Caribe em Ciências da Saúde.

Nineteen references were added in this review (Fig. 1). Of those, 8 (42%) were opinions, letters or short communications; 4 (21%) were reviews; 3 (15%) were official health administration documents from Brazil; 2 (10%) were articles and 2 (10%) others were editorials.

**Figure 2:**
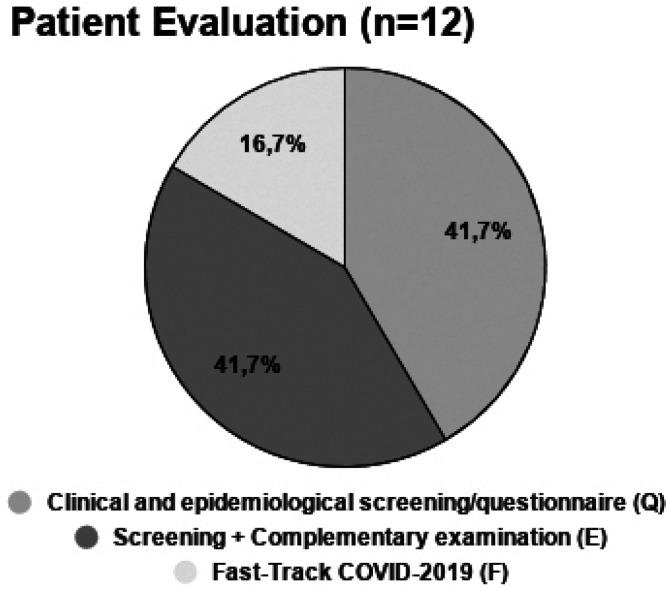
Patient evaluation before dental consultation. Complementary examination mainly consists of temperature assessment. Fast-Track COVID-2019 is one recommended strategy adopted in Brazilian guidelines, in which patients are evaluated by a designated professional before any procedure.

Almost all references suggested only maintaining urgency treatments in positive epidemiological areas for the COVID-19 disease (1-13). Only one article suggested to evaluate with each patient and decide if even non-emergency proceedings should be done (14). In relation to patient evaluation before procedures, all articles that mentioned this approach, 72,2% (n=13), suggested either an epidemiological and clinical questionnaire or a simple clinical exam (Fig. 2).

**Figure 3:**
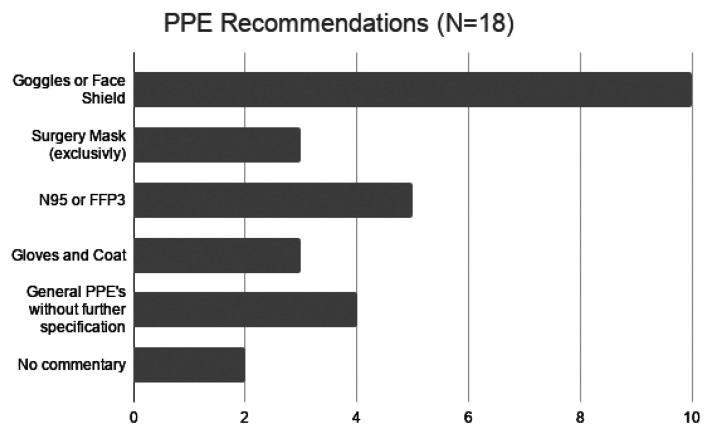
PPE Recommendations for Dentists. PPE=personal protective equipment.

Many references also suggested strict infection control actions for the dental office. Those are grouped in Table 1 (2-18). Regarding mouthrinse, 3 references suggested oxidative agents, 1 suggested chlorhexidine (CHX), essential oils, and cetylpyridinium, chloride (CPC), 2 suggested povidone iodine and 3 suggested the use but did not specify which class (1-3,14). Despite those general orientations, there were also more specific recommendations for the use of Personal Protective Equipment (PPE) by 94,7% (n=18) references (Fig. 3).

**Table 1:**
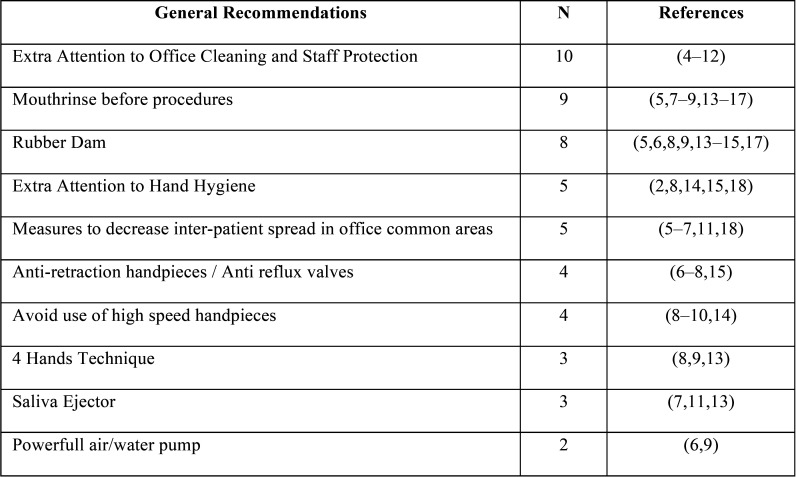
Recommendations for the Dental Office.

## Discussion

The experience of other countries, mainly China, where the COVID-19 outbreak largely affected all social and productive activities, led to the issuing of recommendations for dentists. The reflections regarding dentistry revealed that there is not a real consensus on the provision of dental care during the epidemic of COVID-19, however, some measures are important to protect both patients and professionals. In Brazil the pandemics curve, at present, is still rising and it did not reach its peak of contaminated persons (19). The Brazilian Health Ministry issued a shortlist of measures to be applied during dental assistance, and this workflow has been published in their bulletin and website. This recommendation list does not deal with all aspects of the pandemic’s intersections with dentistry, since most mechanisms and consequences of the infection are still largely unknown.

The American Dental Association (ADA) has been proving to be one of the most active organs in positioning dental practitioners onto the procedures and consequences of assisting patients during the pandemics. This information is added to the recommendations of a number of articles published on the subject (20).

Most publications agree that it is imperative that patients are monitored for COVID-19 infection prior to admission to avoid professionals exposure (13,16,18,21). A precheck triages to measure and record the temperature of every patient must be performed as a routine procedure, using a contact-free forehead (1).

A questionnaire should be used to screen patients with potential infection prior to any procedure (WHO 2020a). The questions most commonly applied to patients are depicted in Table 2. Additionally, patients should be scheduled allowing a large appointment, in order to avoid waiting room overcrowding, and later, a proper office decontamination procedure. Another measure to minimize infection is to postpone health activities in groups such as public campaigns of supervised tooth brushing and Fluor-gel application (21).

**Table 2:**
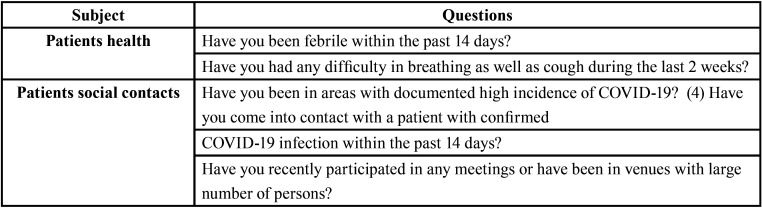
Pre-check questionnaire (2).

The routine practice of dentistry produces high amounts of droplets and aerosols. In this view, professionals should take measures to avoid or minimize procedures in order to reduce this type of risk. The use of saliva ejectors with low or high volume can reduce the production of droplets and aerosols, and the use of rotatory equipment and ultrasonic should be avoided. Additionally, the 4-handed technique is considered important in the control of infection (1,2,6,7,11).

In case of a positive response to any of these questions and body temperature above 37.3oC patient should be advised to self-quarantine and the dental professional should report the case to the health authorities. In case of negative responses and body temperature below 37.3ºC, patient can be treated under extra protection measures. When body temperature is elevated above 37.3ºC, in any case, patient must be advised to seek further medical care, and there is no consensus to postpone their treatment, especially in the presence of pain. Therefore, it is very important that dentists work together with the medical network (6,8).

Most authors recommend the use of a mouthrinse to reduce microbial concentration in the oral cavity. Chlorhexidine, which is commonly used as mouthrinse in dental practice, may not be effective to kill 2019-nCoV, but investigations are necessary to prove its’ efficacy in this situation. Some authors point out that 2019-nCoV is vulnerable to oxidation, and recommend preprocedural mouthrinse containing oxidative agents such as 1% hydrogen peroxide or 0.2% povidone is recommended, for the purpose of reducing the salivary load of oral microbes, including potential 2019-nCoV carriage (1-3,14). This recommendation lacks consensus and scientific support. Many authors, including the Brazilian Health Ministry recommend the use of rubber dam of dental procedures. A preprocedural mouthrinse would, in theory, be most useful in cases when rubber dam cannot be used (2,14).

Most authors regard the use of goggles and/or face shields as very important to protect dentists during procedures. In fact, this type of protection, as well as masks, gloves and impermeable gowns are essential to avoid cross contamination during any procedure, especially those involving the generation of aerosol (2,6-8,11,21,22). Image exams, especially periapical x-rays should also be avoided to prevent the spread of saliva droplets. In case imaging is necessary, a pan-x-ray of CT scans are desirable. Table 3 summarizes professional emergency care during the pandemics, which is the recommendation of most authors (2,6,7,11,21-22).

**Table 3:**
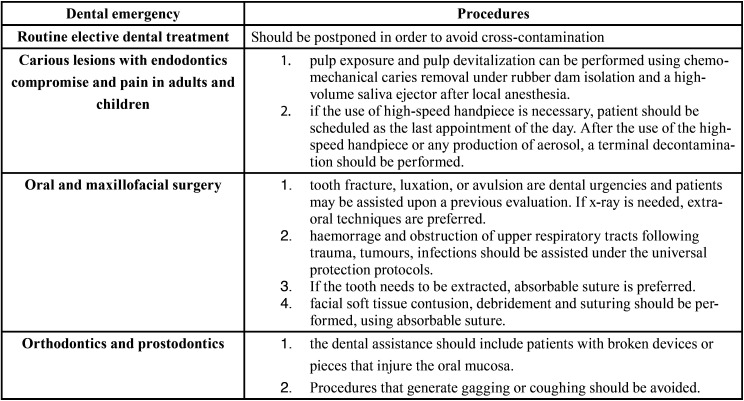
Summary of the main dental emergencies in need of professional assistance during the COVID-19 pandemics (2,6-8,11,13,21,22) .

Finally, special attention is needed for all decontamination procedures in the dental office. Some authors believe that cases suspected or confirmed with COVID-19 should not be treated in the routine dental office, as airborne contamination may occur. Instead, these patients should be referred to dental clinics provided with Negative-pressure treatment rooms. Additionally, the equipment such as the 3-way high-speed rotatory piece, ultrasonic device, and others, should be avoided whenever possible, as they generate high amounts of aerosol. It is well-known that aerosol can spread over the surrounding surfaces and that SARS CoV-2 can remain viable up to 3 days at room temperature (8). Therefore, decontamination of the dental office and surfaces with alcohol at 70%, or 3% sodium hypochlorite, especially when aerosol has been produced is imperative. Removal and decontamination of filters from air conditioning systems such as high-volume evacuator (HVE) or high-efficiency particulate arrestor (HEPA) is an important step to prevent airborne contamination (2,16). The waste generated by the treatment in the dental offices during the pandemics are considered as infectious, in particular when suspected or confirmed cases of COVID19 were assisted. Therefore, a double-layer yellow colour medical waste package bags and “gooseneck” ligation should be identified and used to dispose all infectious material (7).

Supplementary measures to make dental treatment safer should rise from the further understanding of the pandemics, as many recommendations still lack scientific evidence and the quarantine and present social distancing impact the income of all clinics and dental offices. Certainly, dental assistance will, in future re-evaluate all procedures in the dental office, in face of infectious disease, epidemics and pandemics, to provide information for an increasingly better practice of dentistry in hospitals, clinics and faculties.
